# Mechanism research on inhibition of gastric cancer *in vitro* by the extract of *Pinellia ternata* based on network pharmacology and cellular metabolomics

**DOI:** 10.1515/med-2024-1131

**Published:** 2025-02-18

**Authors:** Fan Feng, Ping Hu, Jun Chen, Lei Peng, Luyao Wang, Xingkui Tao, Chaoqun Lian

**Affiliations:** School of Biological and Food Engineering, Suzhou University, Anhui, 234000, China; Research Center of Clinical Laboratory Science, Bengbu Medical University, Bengbu, 233030, China

**Keywords:** gastric cancer, metabolomics, *Pinellia ternata*, network pharmacology, metabolites

## Abstract

**Background and purpose:**

Gastric cancer is a kind of malignant tumor with high incidence and high mortality, which has strong tumor heterogeneity. A classic Chinese medicine, *Pinellia ternata* (PT), was shown to exert therapeutic effects on gastric cancer cells. However, its chemical and pharmacological profiles remain to be elucidated. In the current study, we aimed to reveal the mechanism of PT in treating gastric cancer cells through metabolomic analysis and network pharmacology.

**Methods:**

Metabolomic analysis of two strains of gastric cancer cells treated with the *Pinellia ternata* extract (PTE) was used to identify differential metabolites, and the metabolic pathways were enriched by MetaboAnalyst. Then, network pharmacology was applied to dig out the potential targets against gastric cancer cells induced by PTE. The integrated network of metabolomics and network pharmacology was constructed based on Cytoscape.

**Results:**

The PTE was confirmed to significantly inhibit cell proliferation, migration, and invasion of HGC-27 and BGC-823 cells. The results of cellular metabolomics showed that 61 metabolites were differently expressed in gastric cancer cells of the experimental and control groups. Through pathway enrichment analysis, 16 metabolites were found to be involved in the glycerophospholipid metabolism, purine metabolism, sphingolipid metabolism, and tryptophan metabolism. Combined with network pharmacology, seven bioactive compounds were found in PT, and the networks of bioactive compound–target gene–metabolic enzyme–metabolite interactions were constructed.

**Conclusions:**

In conclusion, this study revealed the complicated mechanisms of PT against gastric cancer. Our work provides a novel paradigm to identify the potential mechanisms of pharmacological effects derived from a natural compound.

## 1 Introduction

Gastric cancer is a malignant tumor originating from the gastric mucosal epithelium, which has highly heterogeneous characteristics. The incidence rate of gastric cancer shows a younger trend due to changes in diet, increased work pressure, and *Helicobacter pylori* infection [1]. According to the latest data from GLOBOCAN 2020, gastric cancer accounts for 7.7% of 10 million cancer deaths worldwide, ranking the fourth cause of death after lung cancer, colorectal cancer, and liver cancer [2]. Despite the numerous advances in targeted therapy for gastric cancer, the overall long-term outcomes of patients have not significantly improved [3,4,5,6]. A large number of studies have found that the components of traditional Chinese medicine (TCM) have the effects of inducing cell apoptosis and inhibiting cell proliferation, and then it can reduce symptoms, inhibit tumor development, and prolong the survival to improve the quality life of patients [7,8,9]. Most currently available chemotherapy drugs, including paclitaxel, vincristine, and vinblastine, are all derived from natural sources [10,11]. Therefore, the clinical prevention and treatment of gastric cancer utilizing the multi-component combination of TCM has garnered research attention.

*Pinellia ternata* (PT), which belongs to the Araceae family, has been widely used in China, Korea, and Japan as a valuable medicinal plant [12]. Modern pharmacological research has demonstrated that PT contains a large number of alkaloids, iridoids, iridoid glycosides, anthraquinones, anthraquinone glycosides, fatty acids, and their derivatives [13,14]. Pharmacological studies have demonstrated that PT has multiple activities, especially antitumor activity [15,16]. Numerous clinical Chinese medicine treatments have shown that PT can inhibit the proliferation and migration of gastric cancer cells, thereby alleviating the symptoms of gastric cancer patients [17]. However, the specific mechanism by which these components inhibit the proliferation of gastric cancer cells remains unclear. Unlike western medicine’s “one target, one drug” concept, TCM emphasizes the concept of the integrity of the whole human body [18]. Because of its complexity in composition, conventional pharmacological approaches to experimentally identify the unique action of mechanism may not be applicable to TCM research [19]. Network pharmacology meets the key ideas of the holistic philosophy of TCM. As a state-of-the-art technique, this method updates the research paradigm from the current “one target, one drug” mode to a new “network target, multicomponents” mode. Nonetheless, network pharmacology is limited by the single computational methods that rely on public databases [20]. Network pharmacology alone could only predict the possibility of compound–target combination and pathway analysis.

Along with the rapid development of bioinformatics, the newly emerging metabolomics based on big databases has become a useful tool to characterize the action mechanisms of complicated drug system in detail, from the molecular level to the pathway level [21,22,23,24]. Metabolomics has been employed in many fields, including the diagnosis and treatment of diseases, biomarker discovery, and exploration of disease pathogenesis [25,26,27]. Numerous studies have shown that metabolomics integrated network pharmacology is an effective method for studying the mechanism of action of TCM [28,29,30]. Therefore, we integrated metabolomics with network pharmacology for analyzing the mechanism of action of PT. This strategy is expected to help better understand the therapeutic principles of natural compounds like PT used in the treatment of gastric cancer.

**Figure 1:**
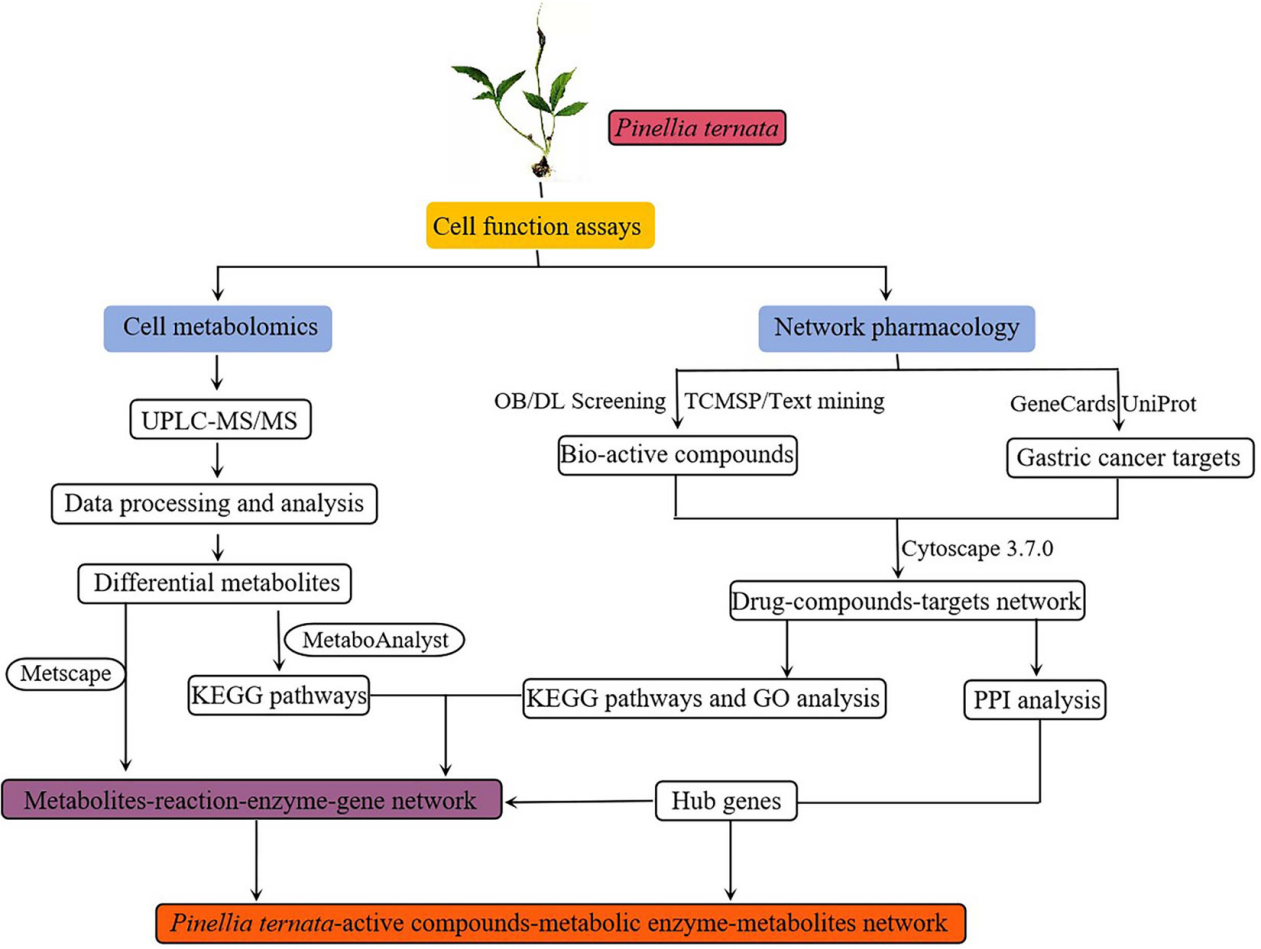
Technical strategy of the current study.

In the current study, we used computational tools and resources to investigate the effect of the pharmacological network of PT on gastric cancer to predict its active compounds and potential protein targets and pathways. In addition, *in vitro* experiments were also conducted to validate the potential underlying mechanism of *PT* in gastric cancer, as predicted by the network pharmacology approach. Meanwhile, a gastric cancer cell metabolomic analysis was performed to reveal the synergetic metabolic mechanisms in terms of metabolites and metabolic pathways. Subsequently, the targets from network pharmacology and the metabolites from cell metabolomics were jointly analyzed to filter crucial metabolism pathways using MetScape. The detailed technical strategy of the current study is shown in Figure 1.

## 2 Materials and methods

### 2.1 Cell experiments

#### 2.1.1 Reagents

The tubers of *PT* were collected from the experimental field at Suzhou University, which was authenticated by Professor Jianping Xue at the College of Life Sciences, Huaibei Normal University. Chemical reagents such as methanol and ethanol (analytical grade) were purchased from China National Pharmaceutical Group Co., Ltd.

#### 2.1.2 Preparation of herb extracts

The extract of *Pinellia ternata* (PTE) was prepared as follows. Two kilograms of *PT* tubers were soaked in 70% ethanol (1:8, w/v) overnight, and the supernatant was collected by centrifugation at 5,000 rpm. The precipitate was extracted twice with 70% ethanol for 2 h, and the supernatant was collected and combined with the previously obtained supernatant. Later, the extract was vacuum-concentrated and freeze-dried into powder and stored at −80°C (the drug extract ratio was 13.8%).

#### 2.1.3 Cell culture and cell viability determination

Human gastric cancer cells HGC-27 and BGC-823 were chosen in our lab for the following experiments. The cells were cultured in Dulbecco’s modified Eagle’s medium (DMEM) supplemented with 10% fetal bovine serum, 100 U/mL penicillin, and 100 mg/mL streptomycin and maintained at 37°C in a humidified chamber with 5% CO_2_.

The human gastric cancer cells (5,000 cells/well) were inoculated into 96-well plates and incubated for 24 h. After pretreatment with different concentrations of PTE (0, 0.0125, 0.025, 0.05, 0.10, 0.20, 0.40, and 0.80 μg/μL) for 48 h, 10 μL of 3-(4,5-dimethylthiazol-2-yl)-2,5-diphenyltetrazolium bromide (MTT, 5 mg/ml; Sigma, USA) solution was added to each well, and then the cells were cultured at 37°C for another 4 h. Then, the supernatants were discarded, and 100 μL of DMSO was added to each well. The absorbance was measured at 490 nm using a Multiskan MS microplate reader (Labsystems, Finland). The half-maximal inhibitory concentration (IC_50_) of gastric cancer cells treated with PTE was calculated by using the GraphPad Prism software.

#### 2.1.4 Cell wound-healing and transwell invasion assays

For the transwell invasion assay, Millicell cell culture inserts in 24-well plates were pretreated with 100 μL of cold Matrigel (BD Biosciences, USA, diluted 1:4 with cold PBS) for 2 h at 37°C. Gastric cancer cells (1 × 10^5^ cells/well) were seeded into the chamber with 200 μL of serum-free DMEM and then incubated with or without PTE at 37°C for 24 h. The invaded cells were fixed with 4% paraformaldehyde for 30 min, stained with crystal violet solution for 2 h, and then counted with a light microscope.

For the wound-healing assay, gastric cancer cells were incubated in 6-well plates with 100% confluence. The denuded area was scrapped using a plastic pipette tip on the cell monolayer. The medium was removed, and the monolayer was washed three times with PBS. Then, the medium with or without PTE was added to each well, and cell movements in the wound area were obtained after 24 h of incubation with a microscope.

### 2.2 UPLC–MS metabolomics analysis

#### 2.2.1 Experimental grouping and sample preparation

The HGC-27 and BGC-823 cells were each cultured in 100 mm cell Petri dish separately and cultured overnight to make the cells to adhere to the wall. The cells were divided into a blank control group and an intervention group, treated with PTE at a concentration 0 or IC_50_, with six parallel samples in each group. Therefore, the treated HGC-27 and BGC-823 cells were divided into four groups: Control-H, PTE-H, Control-B, and PTE-B. After 48 h, the Petri dish was washed three times with precooled PBS and then digested with trypsin for 1–2 min. The suspension was centrifuged at 1,000 rpm for 5 min, the supernatant was discarded, and the cells were collected as samples.

The samples stored at −80°C were thawed on ice. A 500 μL solution (methanol:water = 4:1, v/v) containing internal standard was added into the cell sample and vortexed for 3 min. The sample was placed in liquid nitrogen for 5 min and on dry ice for 5 min, and then thawed on ice and vortexed for 2 min. This freeze–thaw cycle was repeated three times in total. The sample was centrifuged at 12,000 rpm for 10 min (4°C). Then, 300 μL of supernatant was collected and stored at −20°C for 30 min. The sample was then centrifuged at 12,000 rpm for 3 min (4°C). About 200 μL aliquots of supernatant were transferred for LC–MS analysis. The pooled quality control (QC) samples were made by mixing 10 μL aliquots of each sample (one per six samples).

#### 2.2.2 UPLC–QTOF-MS analysis

All samples were acquired by the LC–MS system following the manufacturer’s instructions. The analytical conditions were as follows: UPLC: column, Waters ACQUITY UPLC BEH C18 1.8 µm, 2.1 mm × 100 mm; column temperature, 40°C; flow rate, 0.4 mL/min; injection volume, 2 μL; and solvent system, water (0.1% formic acid):acetonitrile (0.1% formic acid). The column was eluted with 5% mobile phase B (0.1% formic acid in acetonitrile) at 0 min, followed by a linear gradient to 90% mobile phase B (0.1% formic acid in acetonitrile) over 11 min, held for 1 min, then returned back to 5% mobile phase B within 0.1 min, held for 1.9 min, and then rapidly returned to starting conditions.

The data acquisition was performed in the information-dependent acquisition (IDA) mode using Analyst TF 1.7.1 software (Sciex, Concord, ON, Canada). The source parameters were set as follows: ion source gas 1 (GAS1), 50 psi; ion source gas 2 (GAS2), 50 psi; curtain gas (CUR), 35 psi; temperature (TEM), 550°C or 450°C; declustering potential, 60 V or −60 V in positive or negative mode, respectively; and ion spray voltage floating, 5,000 V or −4,000 V in positive or negative mode, respectively.

#### 2.2.3 Data analysis

The original data file acquired by LC–MS was converted into mzML format using ProteoWizard software. Peak extraction, peak alignment, and retention time correction were performed using the XCMS program. The “SVR” method was used to correct the peak area. The peaks with detection rate lower than 50 % in each group of samples were discarded. After that, metabolic identification information was obtained by searching the laboratory’s self-built database, integrated public database, AI database, and metDNA. SIMCA-P 14.1 software (Umetrics, Sweden) was used to conduct principal component analysis (PCA), partial least-squares discriminant analysis (PLS-DA), and orthogonal partial least-squares (OPLS) analysis of the normalized data. Based on variable important in projection (VIP) values (VIP > 1) and *T*-test (*p* < 0.05), the differential abundant metabolites were selected between the control group and the model group and identified according to the online databases: mzCloud (https://www.mzcloud.org/), HMDB (http://www.hmdb.ca), ChemSpider (http://www.chemspider.com), and KEGG (http://www.kegg.jp) [31]. The Venn diagram was drawn according to the guidance method of online software (https://cloud.metware.cn/#/user/login). Pathway analysis was conducted with MetaboAnalyst [32]. Parameters (*p* value <0.05) were used as the index to determine the most relevant pathways.

### 2.3 Network pharmacology analysis

#### 2.3.1 Bioactive component screening

The information on compounds present in PT was obtained from databases such as TCMSP (http://tcmspw.com/) [33]. The active compounds were filtered by integrating OB (≥30%) and DL (≥0.18) [34]. In addition, compounds with definite pharmacological effects were also selected for further research, even though they have low OB or DL values.

#### 2.3.2 Target protein prediction of drug components in PT

The protein targets of the active substances in PT were retrieved from the TCMSP database by using the filter search bar of the related targets of the compound component. Meanwhile, the annotated genome database platform GeneCards, the protein database UniProt, and the online database KOBAS were used to query the human gene names corresponding to the target proteins.

#### 2.3.3 Construction of the protein–protein interaction (PPI) network and screening of its core targets

For construction of the PPI and screening, the following steps were carried out. Log in to the String database online, find the search mode “multiple protein” box, enter the common target proteins of PT and gastric cancer into the String database (https://string-db.org/Version 10.5), and select human (*Homo sapiens*) as search species condition, convert the common target name, set the PPI score to >0.7, obtain the visual interaction map of the PPI network, manually hide the free protein that appears outside the network, and export the PPI relationship map. According to the node degree value, the key core genes of the PPI network were screened out.

#### 2.3.4 Pathway enrichment analysis

To explore the combination mechanisms of PT against gastric cancer, pathway enrichment analysis was performed using the DAVID Bioinformatics Resources 6.8 server [35], and GO and KEGG pathway enrichment analyses of drugs–key chemical components–disease targets were carried out. Pathways with *p* values less than or equal to 0.05 were selected.

#### 2.3.5 Network construction

Combined with the identification and screening of drug target proteins in Section 2.3.3, the gastric cancer target proteins were mapped to each other to obtain common target proteins, and then the related information about the drug active ingredient and the common target proteins was imported into Cytoscape 3.7.1 software for data processing. The visual network of drugs–bioactive components–disease targets was constructed and obtained. Among them, nodes were used to represent key chemical components and disease targets, and solid lines with arrows were used to represent the interaction between nodes.

#### 2.3.6 Joint pathway analysis

The targets from network pharmacology and the metabolites from cell metabolomics were jointly analyzed to select crucial metabolism pathways by MetaboAnalyst [36].

### 2.4 Statistical analysis

Each independent experiment was repeated at least three times. Statistical analysis between two groups was performed by using Student’s *t* test through SPSS software (SPSS Inc., USA). Variance analysis between multiple groups followed by Tukey’s test was used to calculate the statistical significance of the differences. Multiple groups of normalized data were analyzed using one-way ANOVA. Data were shown as mean ± standard deviation. If not specified above, a *p*-value of less than 0.05 was considered to indicate a statistically significant difference.

## 3 Results

### 3.1 Inhibitory effect of PT on the proliferation of gastric cancer cells

**Figure 2:**
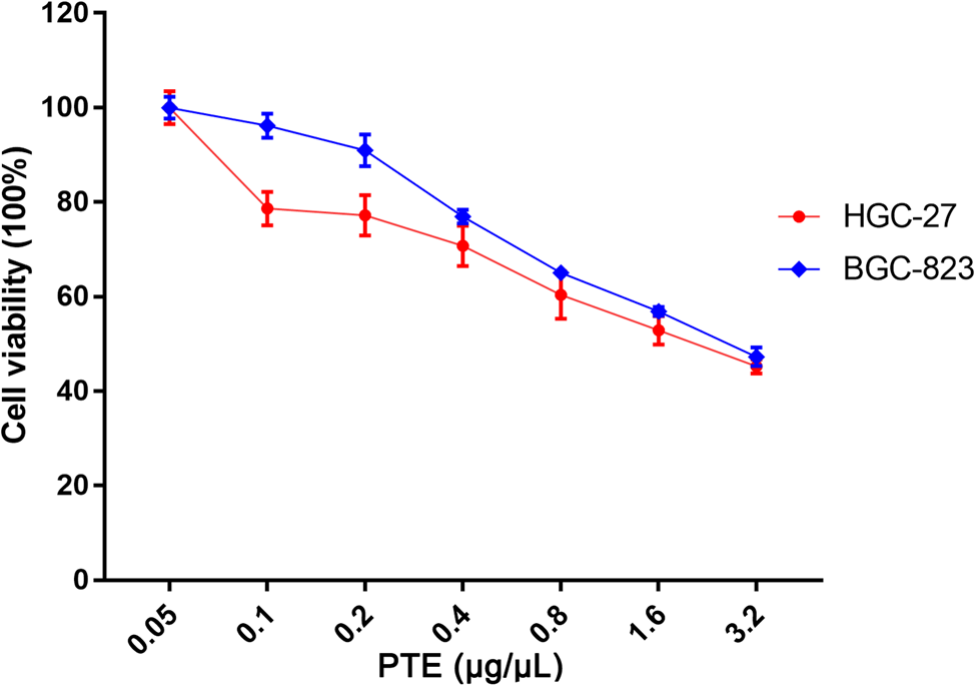
Effects of PTE on the viability of HGC-27 and BGC-823 gastric cancer cells at different concentrations.

To verify the antiproliferative effect of PTE on gastric cancer cells, HGC-27 and BGC-823 cells were each treated with PTE at different concentrations for 48 h (Supplementary File S1). The results showed that the inhibition rate of HGC-27 and BGC-823 cells significantly increased with the increase of PTE concentration, indicating that PTE had a significant inhibitory effect on the growth of gastric cancer cells (Figure 2). The IC_50_ values of HGC-27 and BGC-823 cells treated with PTE were calculated to be 1.76 and 2.20 μg/μL, respectively. For the convenience of subsequent experiments, 1.76 μg/μL PTE was selected for treatment of both cell lines.

### 3.2 PT inhibits the invasion and migration of gastric cancer cells

**Figure 3:**
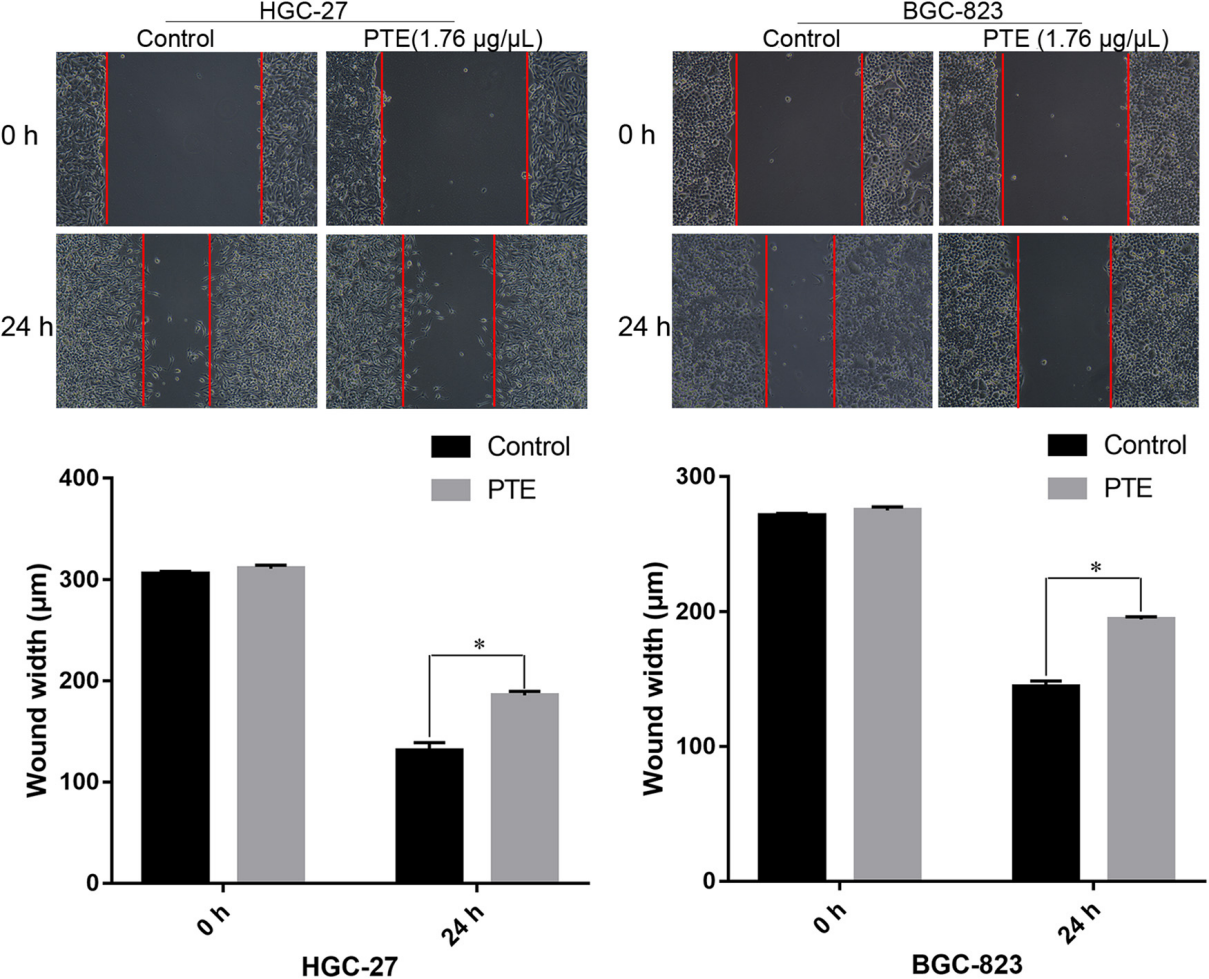
Effects of PTE on the migration of HGC-27 and BGC-823 cells. * represents *p* < 0.05 compared with the control group.

**Figure 4:**
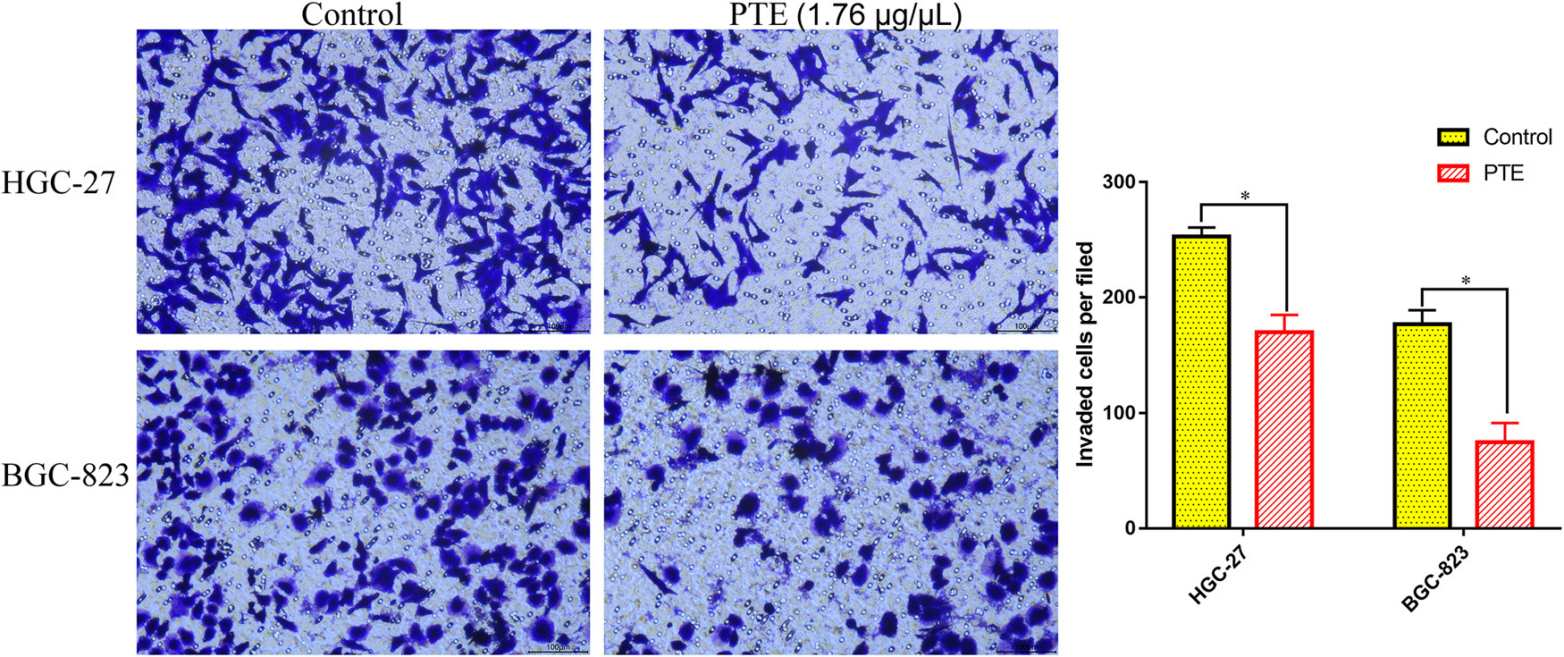
Effects of PTE on the invasion of HGC-27 and BGC-823 cells. * represents *p* < 0.05 compared with the control group.

In order to investigate the effects of PTE on gastric cancer cells cultured *in vitro*, cell migration and invasion experiments were conducted. As shown in Figure 3, the wound-healing assay indicated that the speed of migration was slower in HGC-27 and BGC-823 cells treated with PTE and showed that it could obviously inhibit gastric cancer cell migration at 1.76 μg/μL concentration. In addition, compared with control groups, the speed of invasion was slower in HGC-27 and BGC-823 cells treated with PTE (Figure 4). These experiments indicated that the PTE could significantly inhibit cell migration and invasion *in vitro*.

### 3.3 Results of metabolomics analysis in gastric cancer cells treated with PT

#### 3.3.1 Multivariate data analysis

**Figure 5:**
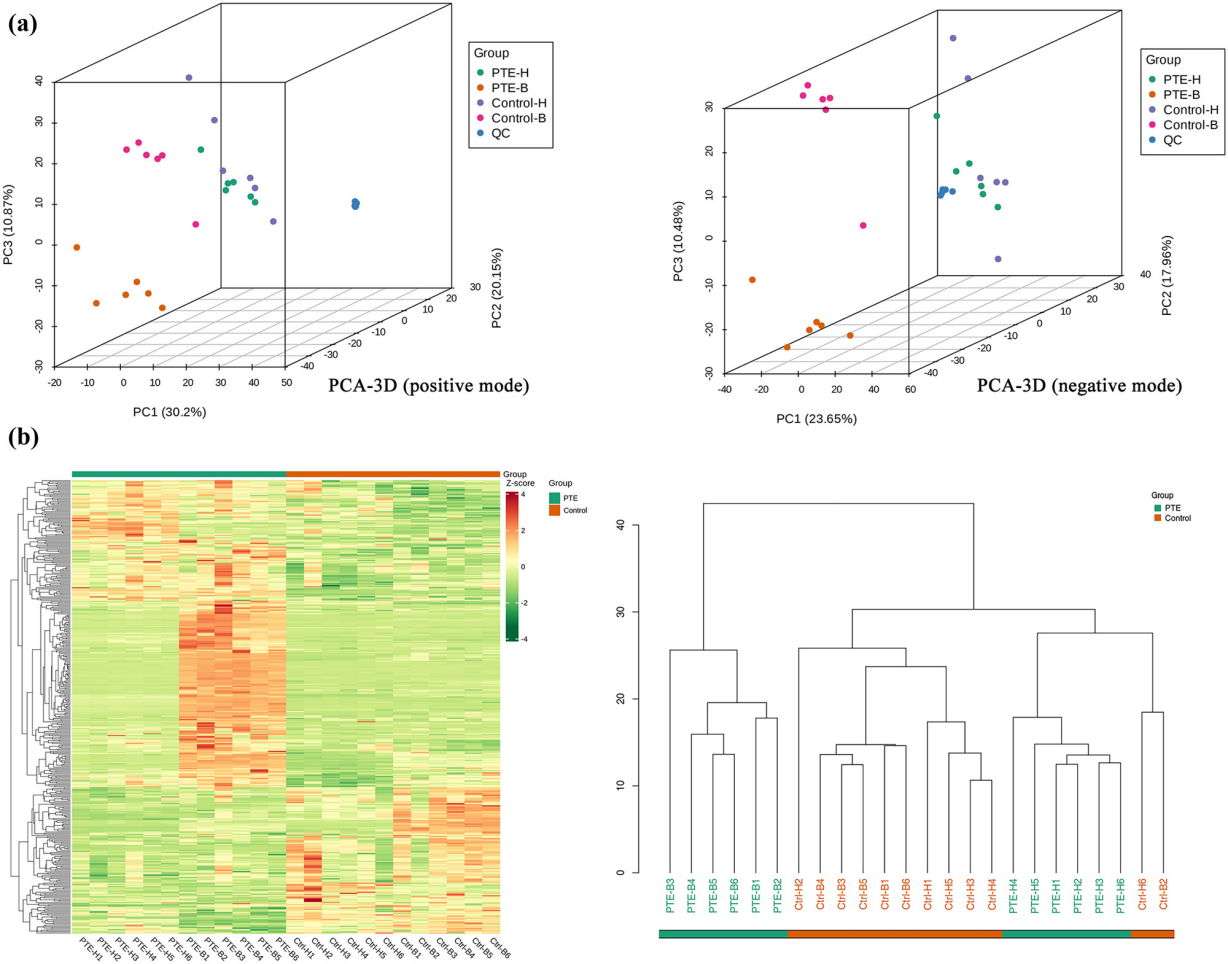
PCA and cluster analysis of overall metabolites. (a) PCA-3D score chart of overall metabolites from LC–MS data in positive and negative modes. (b) Overall cluster diagram of the metabolites. Data were calculated by the Pearson correlation method after mean centering and unit variance scaling.

The typically based peak intensity chromatograms of the HGC-27 and BGC-823 gastric cancer cell samples were analyzed in both negative and positive modes (Figure S1). The PCA score chart indicated that the model group was distinguished from the control group (Figure 5a). Moreover, the QC group was gathered, which indicated that the instrument was stable (Figure 5a). Pearson correlation analysis was conducted on QC samples, and the correlation between QC samples was greater than 0.99 (Figure S2), indicating good stability and high data quality throughout the testing process. As shown in Figure 5a, significant separation between the PTE and control groups was observed in the PCA score 3D plots, indicating that the metabolic disturbances could be obviously induced by PTE treatment. Compared with those in the control group, the metabolic changes in the HGC-27 group were more obvious than those in the BGC-823 group. Further cluster analysis results showed that, after PTE treatment, there were significant differences in the metabolites of different gastric cancer cell lines, with small individual differences within the same group (Figure 5b).

#### 3.3.2 Identification of differential endogenous metabolites

**Figure 6:**
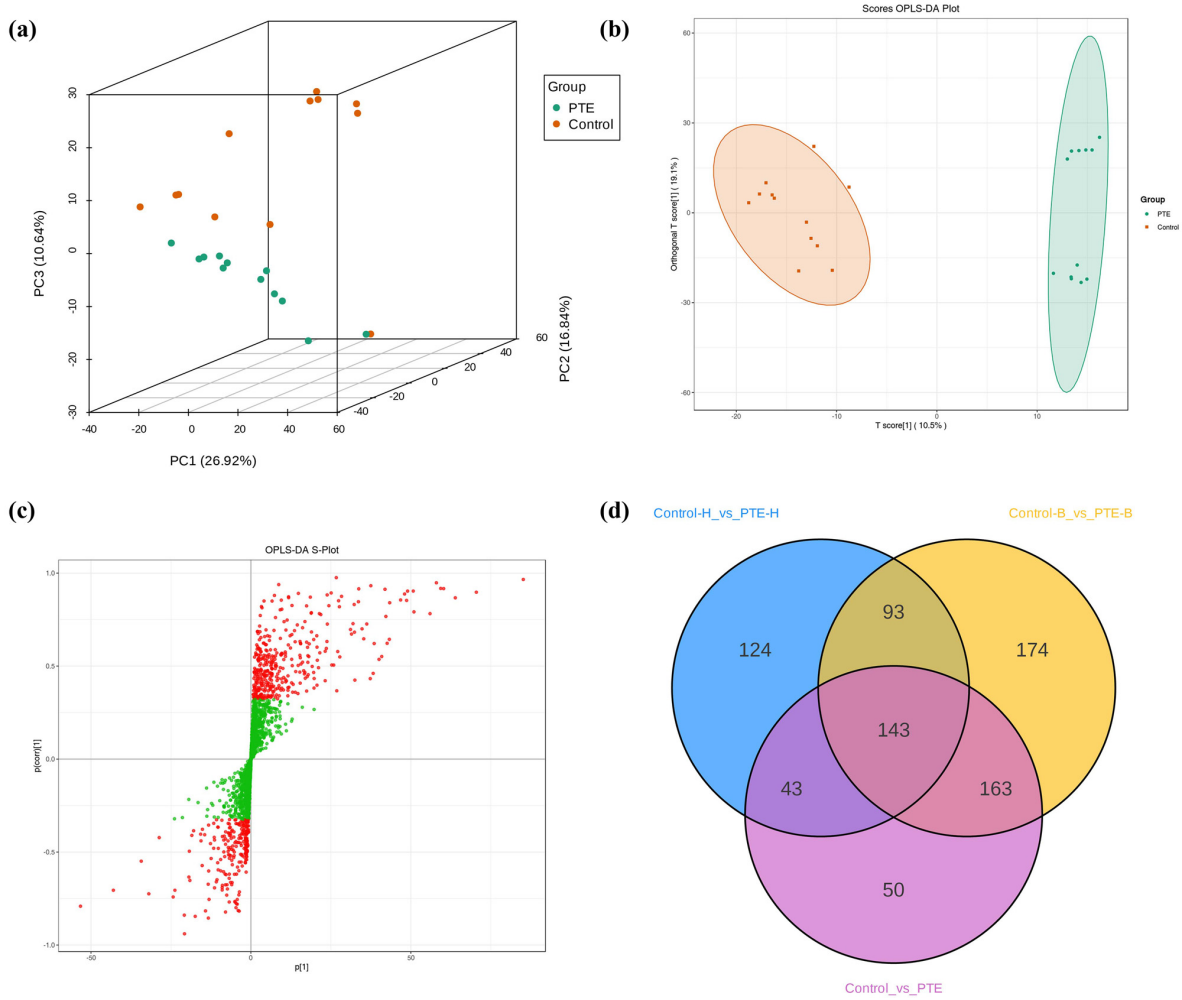
Identification of differential endogenous metabolites. (a) PCA score plots. (b) OPLS-DA score plot. (c) S-plot of OPLS-DA. (d) Venn diagram of the potential metabolites associated with PTE treatment on gastric cancer cells.

After data preprocessing, a total of 4,335 metabolites were identified in gastric cancer cells of the 4 groups (Supplementary File S2). To identify the potential metabolites that contributed to metabolic distinction, we conducted PCA (Figure 6a), OPLS-DA (Figure 6b), and ANOVA, followed by FDR. The OPLS-DA model showed good separability with high *R* ^2^ *Y* (*R* ^2^ *Y* = 0.975, *p* < 0.005) and *Q* ^2^ (*Q* ^2^ = 0.886, *p* < 0.005) values (Figure S3), indicating good explanatory ability of sample classification information and cross-validated predictive capability. The S plots of OPLS-DA were constructed based on the VIP values (Figure 6c), which revealed the variety of metabolites.

Based on VIP > 1 and *p* < 0.05, 403 metabolites in HGC-27 gastric cancer cells were differentially expressed between the PTE-H and Control-H groups, 573 metabolites in BGC-823 gastric cancer cells were differentially expressed between the PTE-B and Control-B groups, and 399 metabolites in gastric cancer cells were differentially expressed between the PTE groups (PTE-H and PTE-B) and Control groups (Control-H and Control-B) (Supplementary File S3-5). Through Venn plot analysis, it was found that there were 143 common metabolic differences between the PTE-treated gastric cancer cell group and the control group (Figure 6d).

#### 3.3.3 Metabolic pathway analysis

**Figure 7:**
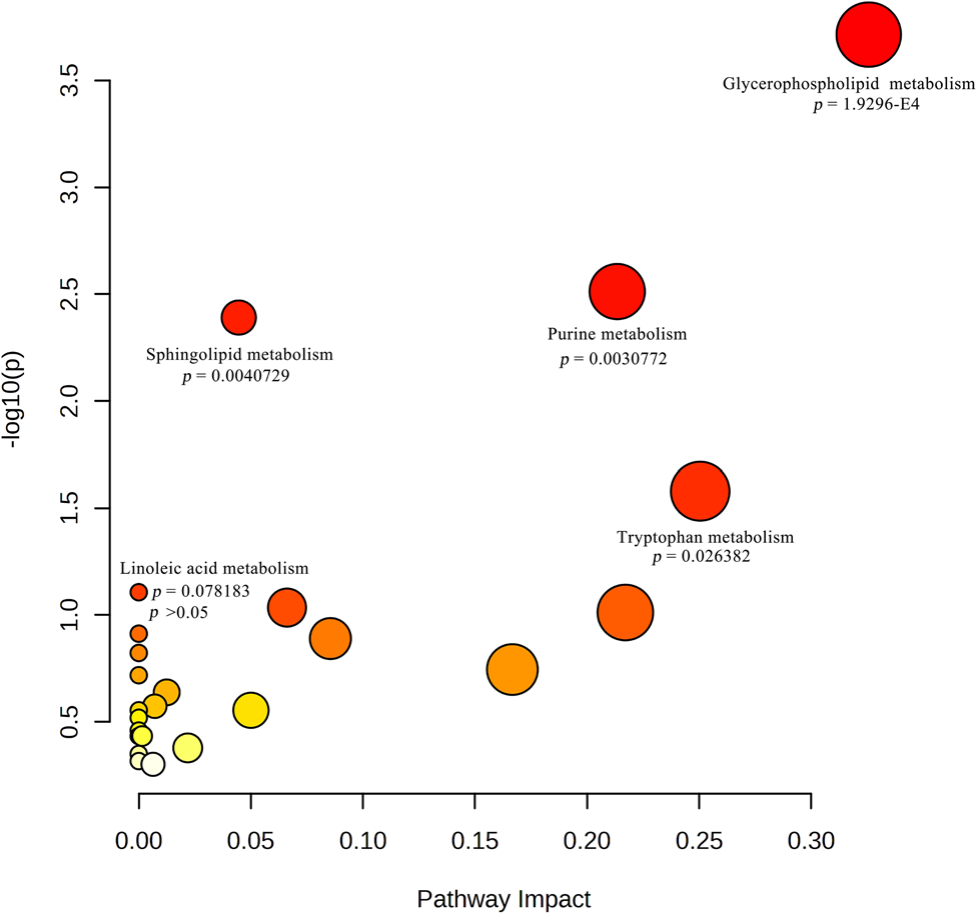
Metabolic pathway analysis. The horizontal axis represents the influence value of the pathway and the vertical axis represents the significance impact value of the signal pathway.

The 143 differential metabolites were imported into KEGG compound database to search their matching KEGG ID, and only 61 metabolites could be identified with the KEGG ID (Supplementary File S6). Then, these metabolites were imported into MetaboAnalyst to explore the potential anti-gastric cancer mechanisms of PT, and 16 metabolites could be matched for further pathway analysis. As shown in Figure 7, based on the *p*-value pathway less than 0.05, four pathways in the gastric cancer cells were significantly affected, including glycerophospholipid metabolism, purine metabolism, sphingolipid metabolism, and tryptophan metabolism (Figure S4). The metabolites related to these pathways were phosphatidylcholine, 1-acyl-sn-glycero-3-phosphocholine, choline, phosphatidate, adenosine monophosphate (AMP), inosine monophosphate (IMP), inosine diphosphate (IDP), sn-glycero-3- phosphocholine, xanthosine, hypoxanthine, sphingomyelin, sphingosine, l-serine, 5-hydroxy-l-tryptophan, l-formylkynurenine, and indoleacetaldehyde (Table 1).

**Table 1:** Differential metabolites related to the inhibitory effect of PT on gastric cancer cells detected by UPLC–MS

No.	Metabolites	TR (min)	*m*/*z*	Formula	VIP	*P*	Fold change	Trend	KEGG ID	Scan mode
1	Phosphatidylcholine	12.35	785.59	C_26_H_52_NO_8_P	1.32	2.71 × 10^−2^	1.36	**↑**	C00157	+
2	1-Acyl-sn-glycero-3-phosphocholine	9.20	579.42	C_30_H_62_NO_7_P	1.39	3.38 × 10^−2^	0.65	**↓**	C04230	−
3	Choline	3.95	393.54	C_20_H_44_NO_4_P	1.51	3.81 × 10^−2^	1.69	**↑**	C00114	−
4	sn-Glycero-3-phosphocholine	0.74	257.10	C_8_H_20_NO_6_P	1.41	4.65 × 10^−3^	1.84	**↑**	C00670	+
5	AMP	1.08	347.06	C_10_H_14_N_5_O_7_P	1.48	3.02 × 10^−2^	1.32	**↑**	C00020	−
6	Phosphatidate	3.09	998.48	C_43_H_85_O_19_P_3_	1.50	5.02 × 10^−3^	1.75	**↑**	C00626	−
7	IMP	0.74	348.05	C_10_H_13_N_4_O_8_P	2.35	5.27 × 10^−6^	1.91	**↑**	C00130	−
8	IDP	0.74	428.01	C_10_H_14_N_4_O_11_P_2_	1.72	1.68 × 10^−2^	1.44	**↑**	C00104	−
9	Xanthosine	1.64	284.07	C_10_H_12_N_4_O_6_	1.89	1.56 × 10^−3^	3.12	**↑**	C01762	−
10	Hypoxanthine	1.08	136.04	C_5_H_4_N_4_O	1.13	4.49 × 10^−2^	1.81	**↑**	C00262	−
11	Sphingomyelin	12.22	812.68	C_47_H_93_N_2_O_6_	1.85	4.26 × 10^−4^	0.72	**↓**	C00550	+
12	Sphingosine	6.17	299.28	C_18_H_37_NO_2_	2.98	6.41 × 10^−4^	3646.44	**↑**	C00319	+
13	l-Serine	0.68	105.04	C_3_H_7_NO_3_	2.63	1.09 × 10^−8^	10.23	**↑**	C00065	−
14	5-Hydroxy-l-tryptophan	2.03	220.08	C_11_H_12_N_2_O_3_	2.54	3.46 × 10^−4^	3.60	**↑**	C00643	−
15	l-Formylkynurenine	2.49	236.08	C_11_H_12_N_2_O_4_	1.21	1.85 × 10^−2^	1.92	**↑**	C02700	+
16	Indoleacetaldehyde	2.39	159.07	C_10_H_9_NO	1.36	1.71 × 10^−2^	1.65	**↑**	C00637	−

### 3.4 Network pharmacological analysis results of PT

A total of 116 chemical components of PT were retrieved from the TCMSP database by keyword screening. Setting the inclusion criteria as DL ≥ 0.18 and OB ≥ 30%, a total of 13 candidate compounds were retrieved (Table S1). Among them, 24-ethylcholest-4-en-3-one, β-sitosterol, poriferast-5-en-3beta-ol, cavidine, baicalin, stigmasterol, and other components have drug-like properties of more than 75%, suggesting that these chemical components may play a key regulation role in the function of medicine in the human body. A total of 175 human target genes were matched from the 13 compound components searched through the TCMSP database. After deduplication, 99 target genes were finally obtained.

**Figure 8:**
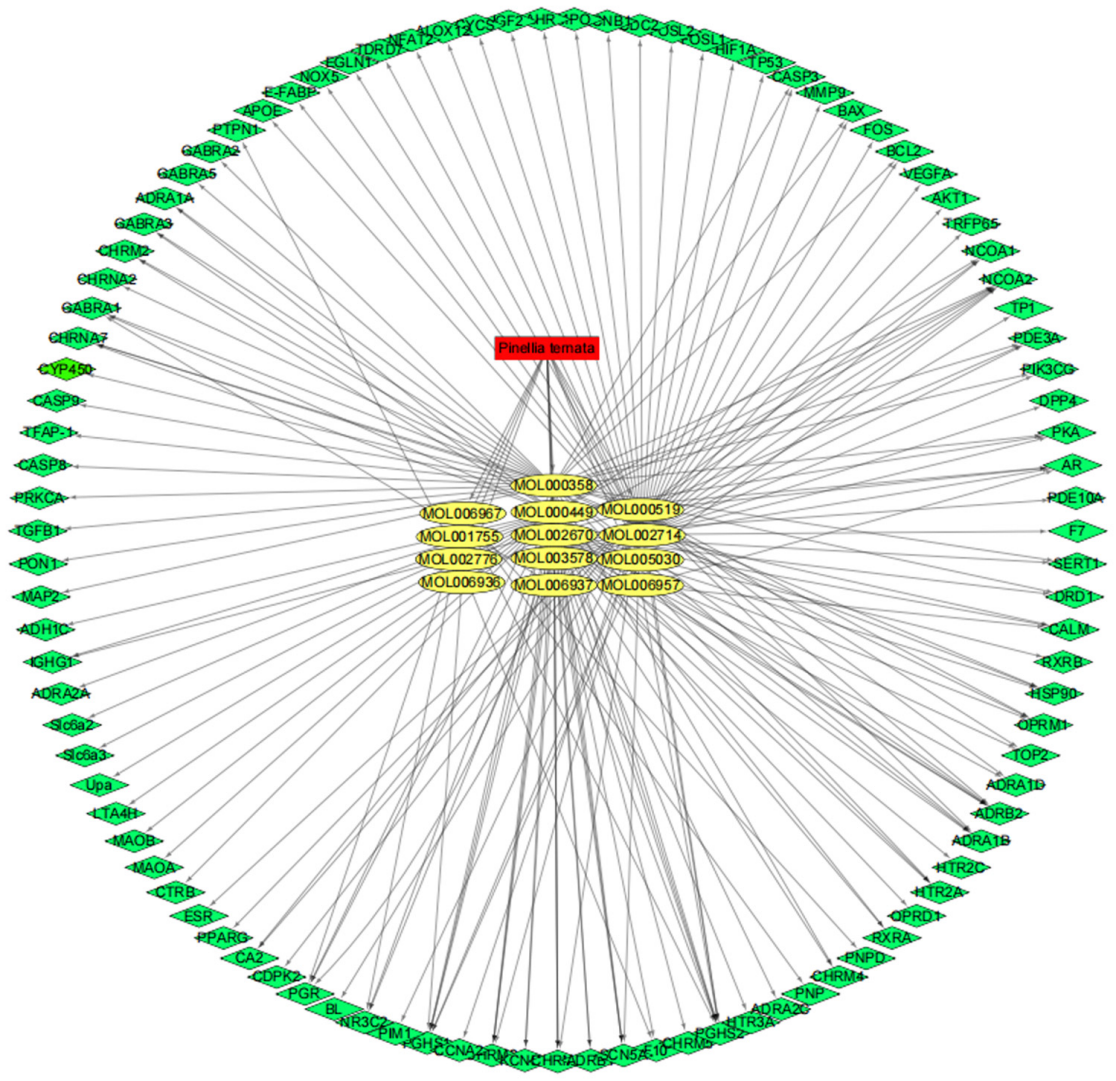
Drugs–bioactive components–disease targets network for PT on gastric cancer. The red nodes represent the PT drug, the yellow nodes represent candidate active compounds, and green nodes represent potential protein targets. The edges represent the interactions between these nodes.

Then, the 13,731 target genes of gastric cancer were obtained from the GeneCards database by using “Gastric cancer” as the screening keyword, then a total of 4,559 genes were obtained by setting the correlation score greater than or equal to 6. Combining the screened 99 drug targets for mutual mapping, 99 common target genes were obtained. The relationship between these 13 compounds and 99 target protein was analyzed by Cytoscape software to construct a network visualization map of drugs–key chemical components–disease targets, as shown in Figure 8.

### 3.5 Integrated analysis of metabolomics and network pharmacology

**Figure 9:**
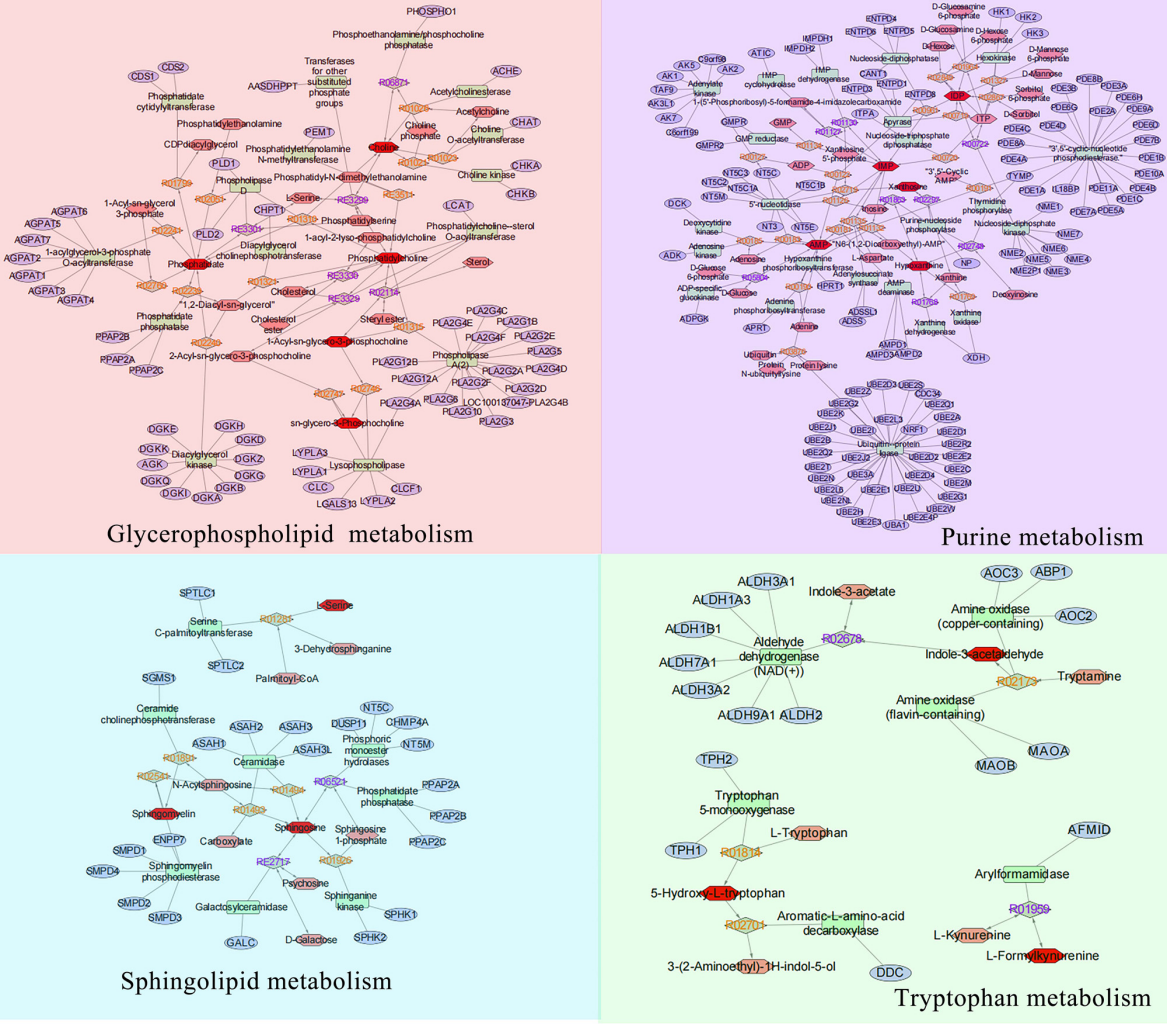
Compound–reaction–enzyme–gene networks of the key metabolites. The red hexagons, gray diamonds, green rectangles, and blue ovals represent the metabolite compounds, reactions, enzymes, and genes, respectively.

To obtain a comprehensive view of the mechanisms of PT against gastric cancer cells, we constructed an interaction network based on metabolomics and network pharmacology. Differentially abundant metabolites were imported into the MetScape plugin in Cytoscape to construct the metabolite–reaction–enzyme–gene networks. As shown in Figure 9, 51 metabolic enzymes associated with 16 differentially abundant metabolites were identified.

**Figure 10:**
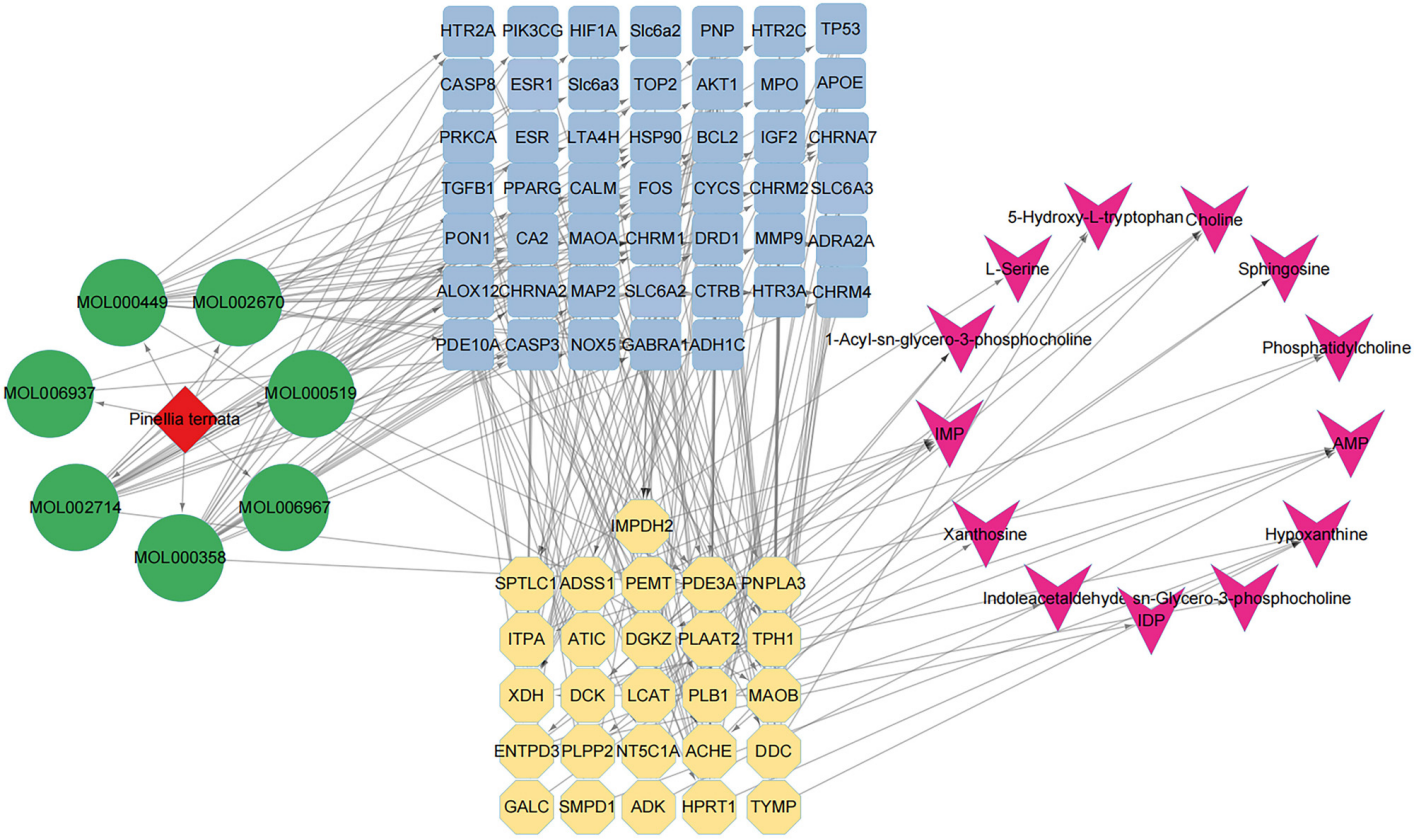
Networks of drug–bioactive components–target genes–metabolic enzymes–metabolites. The red diamond node represents PT drug, green circle nodes represent the candidate active components in PT, blue square nodes represent the target genes of the active components, yellow hexagon nodes represent the key metabolic enzymes, and the purple arrow nodes represent the key metabolites.

To further investigate how the target genes of effective components in PT regulate metabolic enzymes to differentially express metabolites, the above 99 target protein and 51 metabolic enzymes were analyzed for protein interaction via the DAVID database. Through interrelated mappings, 69 target genes of 7 compounds in *PT* were found to be closely related to 26 metabolic enzymes in gastric cancer cells, which produced 13 differential metabolites (Figure 10). The affected pathways were glycerophospholipid metabolism, purine metabolism, sphingolipid metabolism, and tryptophan metabolism. These compounds may play essential roles in the inhibitory effect of PT on gastric cancer cells.

## 4 Discussion

PT, a TCM, has the effect of reducing nausea and vomiting, which can be used to treat vomiting and nausea, protect the gastric mucosa, promote the repair of the gastric mucosa, and have the effect of resisting stress ulcers [37]. Our experiments showed that the PTE could significantly inhibit the proliferation, migration, and invasion of gastric cancer cells *in vitro.* By using network pharmacology methods, 13 ingredients in PT were selected through the TCMSP database, mainly including sterols, alkaloids, flavonoids, and cyprinosides. The results of modern pharmacological research showed that these categories of compounds played an important role in regulating tumorigenesis, suggesting that the network pharmacology for screening effective active ingredients of drugs had important reference values [38]. However, these studies still lack research on the anti-tumor functions and have not systematically identified which components participate in the regulatory mechanisms to affect the malignant phenotype characteristics of cancer from the perspective of metabolic pathways.

Researchers are increasingly relying on metabolomics to explore disease mechanisms and intervention strategies. We identified 16 significant metabolites of PT against gastric cancer cells, as well as their related metabolic pathways. However, given the complexity and heterogeneity of metabolomics, data analysis and interpretation were collaborative efforts [39]. Network pharmacology is a system biology-based methodology [40]. It evaluates drug polypharmacological effects at a molecular level to predict the interaction of natural products and proteins as well as to determine the major mechanisms [41]. Network pharmacology can further validate the therapeutic regulation of metabolic networks and facilitate the identification of key targets and biomarkers [42]. In this study, network pharmacology greatly improved the screening of metabolites of PT against gastric cancer and explicated the action mechanisms. By combining metabolomics with network pharmacology, we found 7 bioactive compounds, 69 key targets, 26 metabolic enzymes and 13 metabolites (phosphatidylcholine, 1-acyl-sn-glycero-3-phosphocholine, choline, sn-glycero-3-phosphocholine, AMP, IMP, xanthosine, IDP, hypoxanthine, sphingosine, l-serine, 5-hydroxy-l-tryptophan, and indoleacetaldehyde), and 4 related pathways (glycerophospholipid metabolism, purine metabolism, sphingolipid metabolism, and tryptophan metabolism). This strategy provides a suitable method to verify the results of the two approaches. It is also practicable to screen metabolites and targets in other natural compounds.

Phospholipids are mainly divided into two categories: glycerol phospholipids and sphingosine phospholipids, which are the main components of cell membranes. Changes in phospholipid metabolism directly affect cell membrane synthesis and cell proliferation. Multiple phospholipid molecules and their metabolic intermediates can participate in cell signal transduction, inflammation, and vascular regulation and are closely related to cell proliferation, adhesion, and movement [43,44]. Therefore, abnormal phospholipid metabolism is closely related to tumor occurrence, development, invasion, and metastasis. Glycerophospholipid metabolism regulates the metabolites of tumors, mainly including glycerophospholipids, lysophosphatidic acid, choline, phosphocholine, phosphatidate, and phosphatidylcholine. These metabolites can affect the proliferation, differentiation, and apoptosis of tumor cells, thereby affecting the occurrence and development of tumors [45]. By regulating these metabolites, new anti-tumor drugs and treatment strategies can be developed [46]. Sphingolipid metabolism, also known as neural sphingolipid metabolism, plays an important role in regulating tumors. Sphingolipids activate signal transduction by stimulating the formation of cell membrane microdomains, thereby regulating cell proliferation, differentiation, apoptosis, and tumor metastasis. Specifically, some molecules in sphingolipid metabolism, such as sphingosine and sphingosine-1-phosphate, can affect the migration and metastasis of tumor cells [47]. These molecules increase cancer cell migration and metastasis by stimulating intercellular communication within tumors. In addition, these molecules may also affect the survival of tumor cells, affecting their growth and proliferation by regulating their survival signal transduction. Interestingly, converting sphingolipids to glycerophospholipids could facilitate cancer progression in human hepatocellular carcinoma and colon cancer [48]. All these studies suggest that PT may inhibit the proliferation of gastric cancer cells by promoting glycerophospholipid metabolism and sphingolipid metabolism.

Purine metabolism represents a potential therapeutic pathway in cancer therapy. Purine, an abundant substrate in organisms, is a critical raw material for cell proliferation and an important factor for immune regulation [49]. The purine *de novo* pathway and salvage pathway are tightly regulated by multiple enzymes, and dysfunction in these enzymes leads to excessive cell proliferation and immune imbalance that result in tumor progression [50]. For example, inosine strongly enhances the proliferation of human melanoma cells, and the altered ratio of adenosine to inosine has been widely noticed in cancer cells, affecting the growth, invasiveness, and metastasis [51]. Meanwhile, purines serve as potent modulators in the response of immune cells and cytokine release via various receptor subtypes, such as P2X ligand-gated ion channels and G protein-coupled P2Y receptors [52], which are substantially involved in the development of oncogenesis and tumorigenesis [53]. Xanthosine is catalyzed by the substrate xanthine or xanthosine 5’-phosphate through the activity of purine-nucleoside phosphorylase or 5’-nucleotidase. The levels of xanthosine, IDP, IMP, AMP, and hypoxanthine are increased in PTE-treated gastric cancer cell groups. Studies have shown that administration of xanthosine did not affect the proportion of epithelial stem cells in bovine breast tissues but had potential negative effects on cell proliferation, and tumor development in mice was also limited by xanthosine administration [54]. Studies also indicated that 5-aminoimidazole-4-carboxamide riboside combined with methotrexate exerted synergistic anticancer action against human breast cancer and hepatocellular carcinoma [55]. Therefore, PT may inhibit the proliferation of gastric cancer cells through purine metabolism, especially by changing the metabolic levels of xanthosine, IDP, IMP, AMP, and hypoxanthine.

Tryptophan is an essential amino acid available from one’s diet by ingestion of food containing it. About 95% of the free tryptophan in the human body undergoes catabolism through the kynurenine pathway, participating in the regulation of immunity, neuronal function, and intestinal homeostasis [56]. Tryptophan metabolic imbalance has attracted great attention in the treatment of cancer and neurodegenerative diseases, especially targeted the regulation of rate limiting enzymes such as amine oxidase, tryptophan-5-monooxygenase, and arylformamidase [57,58]. In this study, indole-3-acetaldehyde, 5-hydroxy-l-tryptophan, and l-formylkynurenine were found to be abnormally expressed in gastric cancer cells treated with PTE compared with the control group. Indole-3-acetaldehyde was synthesized from the substrate tryptamine through the activity of amine oxidase. 5-Hydroxy-l-tryptophan was synthesized from the substrate l-tryptophan by the activity of tryptophan-5-monooxygenase. l-Formylkynurenine was synthesized from the substrate l-kynurenine through the activity of arylformamidase. Current research has found that there is still a lack of research on the correlation of three metabolites indole-3-acetaldehyde, 5-hydroxy-l-tryptophan, and l-formylkynurenine with tumors. Interestingly, the latest research has found that tryptophan metabolites 5-hydroxytryptamine and 3-hydroxyanthranilic acid could protect tumor cells from iron death and promote tumor growth [59]. This indicates that PT may inhibit the proliferation of gastric cancer cells by promoting tryptophan metabolism.

## 5 Conclusions

In this study, we have demonstrated that PT inhibited the proliferation, migration, and invasion of gastric cancer cells. Subsequently, 13 key metabolites and 4 important metabolic pathways were identified through cell metabolomics screening. Combined with network pharmacology, we identified 7 effective active components, and the association network, PT–bioactive component–target gene–metabolic enzyme–metabolite, was constructed. This is the first development of a new comprehensive strategy based on metabolomics and network pharmacology to explore key targets and mechanisms of PT in the treatment of gastric cancer. This study provides data and theoretical support for in-depth research on its mechanism of action, laying the foundation for clinical applications. Further systematic molecular biology experiments are needed to verify the accurate mechanism. It also provides a new paradigm to determine the potential mechanisms of pharmacological effects of natural compounds.

## Abbreviations

DL: drug likeness
PT: *Pinellia ternata*
OB: oral bioavailability
PPI: protein–protein interaction
TCM: traditional Chinese medicine

## Supplementary Material

Supplementary Table 1

Supplementary Table 2

Supplementary Table 3

Supplementary Table 4

Supplementary Table 5

Supplementary Table 6

Supplementary Figure
