# Supplementary material for: Mechanism research on inhibition of gastric cancer in vitro by the extract of Pinellia ternata based on network pharmacology and cellular metabolomics
Source: Open Med (Wars). 2025 Feb 18;20(1):20241131. doi: 10.1515/med-2024-1131 (PMC11843166; doi:10.1515/med-2024-1131)
Supplement: Supplementary Figure [file med-2024-1131-s7.pdf]

Supplementary material

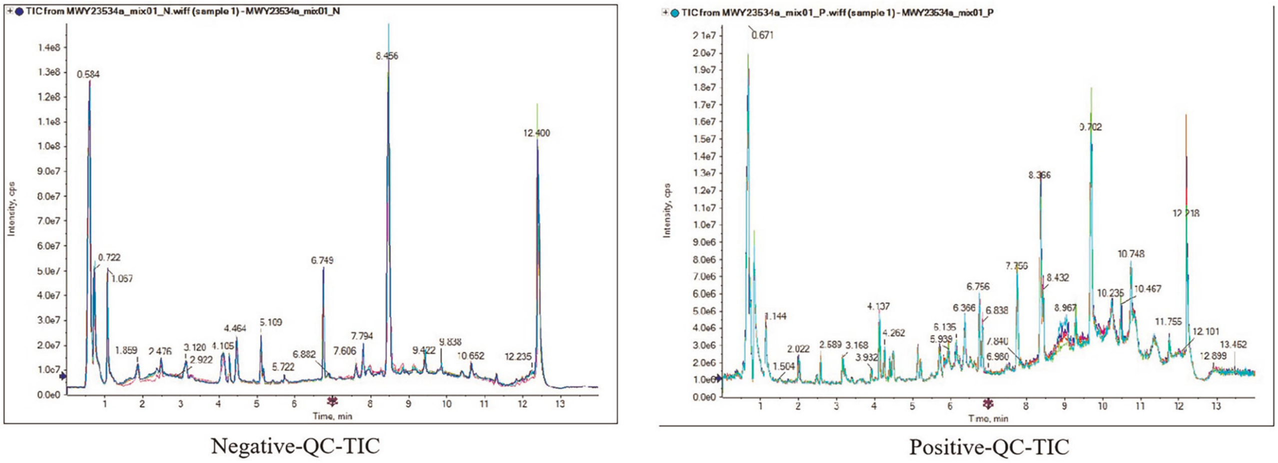

Figure S1: The typically based peak intensity chromatograms of gastric cancer cell HGC-27 and BGC-823 samples were analyzed in both positive and negative modes.

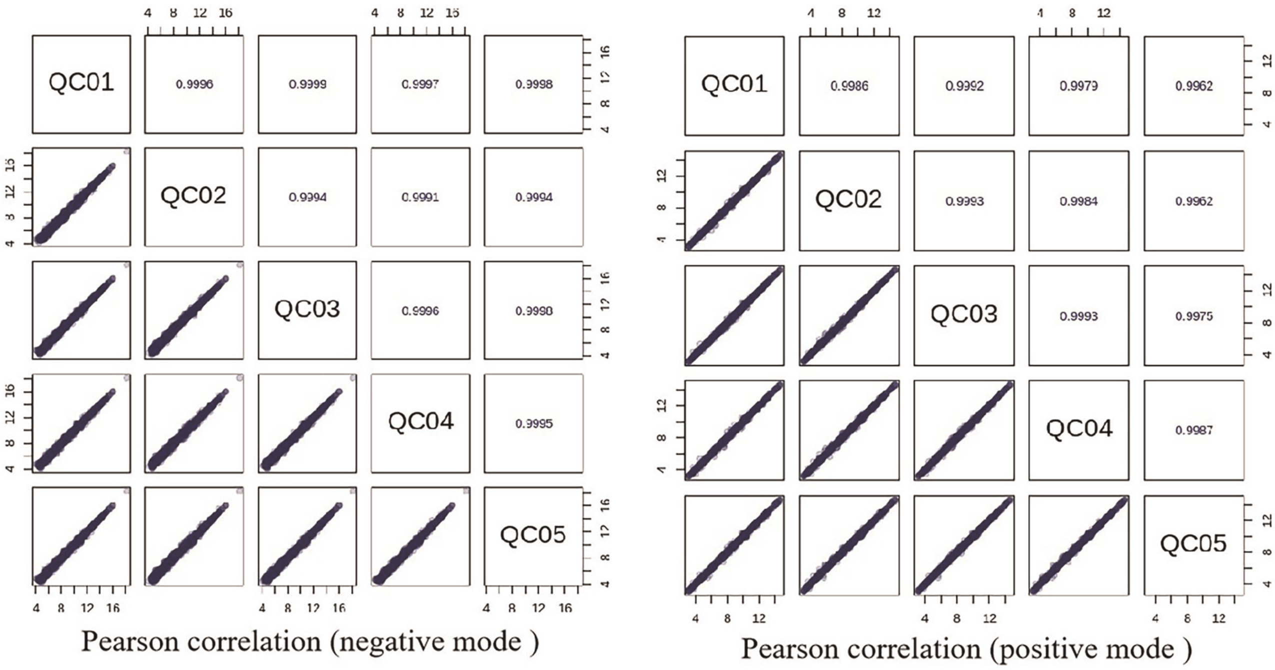

Figure S2: Pearson correlation analysis was conducted on QC samples, and the correlation.

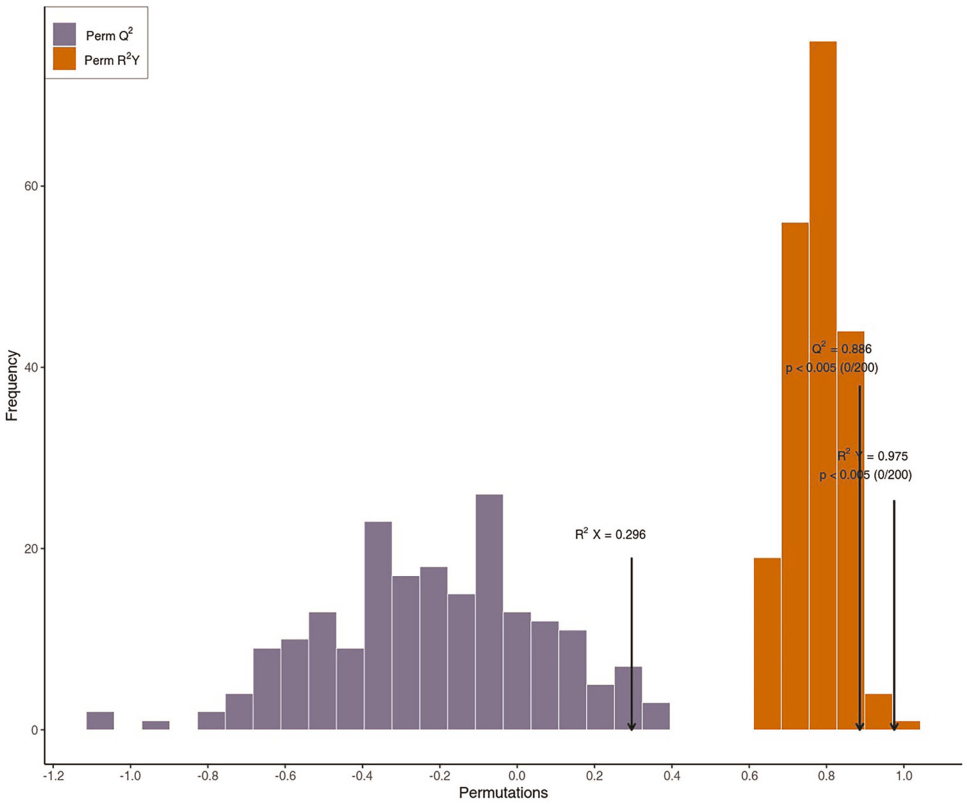

Figure S3: The OPLS-DA model showed good separability with high R^2Y (R^2Y = 0.975, $p < 0.005$) and Q^2 (Q^2 = 0.886, $p < 0.005$).

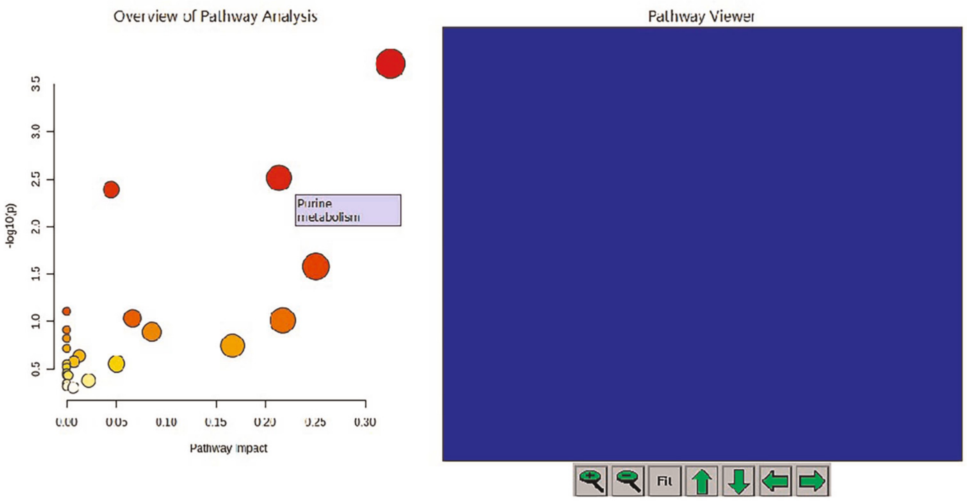

Click the corresponding **Pathway Name** to view its graphical presentation; click **Match Status** to view the pathway compounds (with matched ones highlighted).

Pathway Name	Match Status	p	-log(p)	Holm p	FDR	Impact	Details
Glycerophospholipid metabolism	5/36	1.9296E-4	3.7145	0.016209	0.016209	0.32565	KEGG
Purine metabolism	5/55	0.0030772	2.5118	0.25541	0.11404	0.21345	KEGG SMP
Sphingolipid metabolism	3/21	0.0040729	2.3901	0.33398	0.11404	0.04462	KEGG SMP
Tryptophan metabolism	3/41	0.026382	1.5787	1.0	0.55402	0.25045	KEGG SMP
Linoleic acid metabolism	1/5	0.073183	1.1069	1.0	1.0	0.0	KEGG SMP

Figure S4: Four pathways were affected significantly in the gastric cancer cells, including glycerophospholipid metabolism, purine metabolism, sphingolipid metabolism, and tryptophan metabolism.

Table S1: Basic information of 13 bio-active compounds in *Pinellia ternata*

Number	Molecular name	OB (%)	DL	Molecules structure	Herb
MOL1755	24-Ethylcholest-4-en-3-one	36.08	0.76	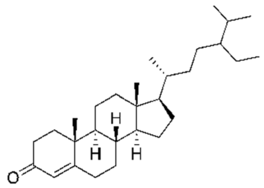	Pinellia
MOL449	Stigmasterol	43.83	0.76	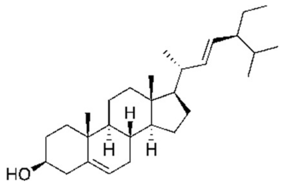	Pinellia
MOL358	beta-sitosterol	36.91	0.75	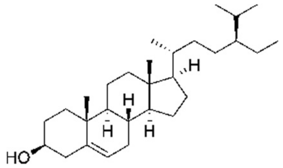	Pinellia
MOL5030	gondoic acid	30.70	0.20	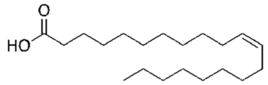	Pinellia
MOL2670	Cavidine	35.64	0.81	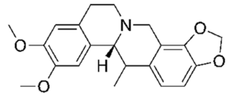	Pinellia
MOL2776	Baicalin	40.12	0.75	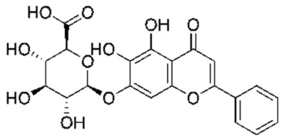	Pinellia
MOL2714	baicalein	33.52	0.21	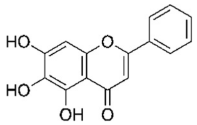	Pinellia
MOL519	coniferin	31.11	0.32	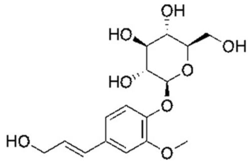	Pinellia
MOL6936	10,13-eicosadienoic	39.99	0.20	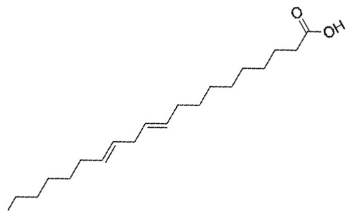	Pinellia
MOL3578	Cycloartenol	38.69	0.78	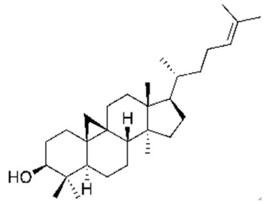	Pinellia

(Continued)

Table S1: *Continued*

Number	Molecular name	OB (%)	DL	Molecules structure	Herb
MOL6957	(3S,6S)-3-(benzyl)-6-(4-hydroxybenzyl)piperazine-2,5-quinone	46.89	0.27	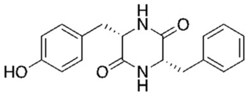	Pinellia
MOL6937	12,13-epoxy-9-hydroxynonadeca-7,10-dienoic acid	42.15	0.24	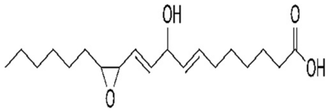	Pinellia
MOL6967	beta-D-Ribofuranoside	44.72	0.21	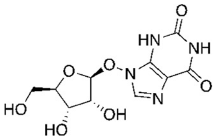	Pinellia

OB, Oral bioavailability; DL, drug-likeness.
